# Search, coagulation, and clipping with the shrink method to minimize ulcer base and prevent delayed bleeding after gastric endoscopic resection

**DOI:** 10.1055/a-2734-0575

**Published:** 2025-11-25

**Authors:** Satoshi Abiko, Yukiko Okada, Kazuki Yamamoto, Yohei Nishikawa, Ippei Tanaka, Haruhiro Inoue, Naoya Sakamoto

**Affiliations:** 1 Digestive Disease Center, Showa Medical University Koto Toyosu Hospital, Tokyo, Japan; 2 378609 Department of Gastroenterology and Hepatology, Hokkaido University Hospital, Sapporo, Japan

## Introduction

Although the search, coagulation, and clipping (SCC) technique has been shown to be more effective than the post-endoscopic submucosal dissection (ESD) coagulation method in reducing delayed bleeding (DB) after gastric ESD 1 , it does not entirely eliminate risk of DB. Applying large-sized clips during clipping may reduce the size of the mucosal defect, which could potentially promote ulcer healing and thereby lead to more efficient prevention of DB. Prevention of DB using the SCC with the shrink (SCC-S) method is reported here.

## Case report

Video showing application of the search, coagulation, and clipping with the shrink method.Video 1

**Fig. 1:**
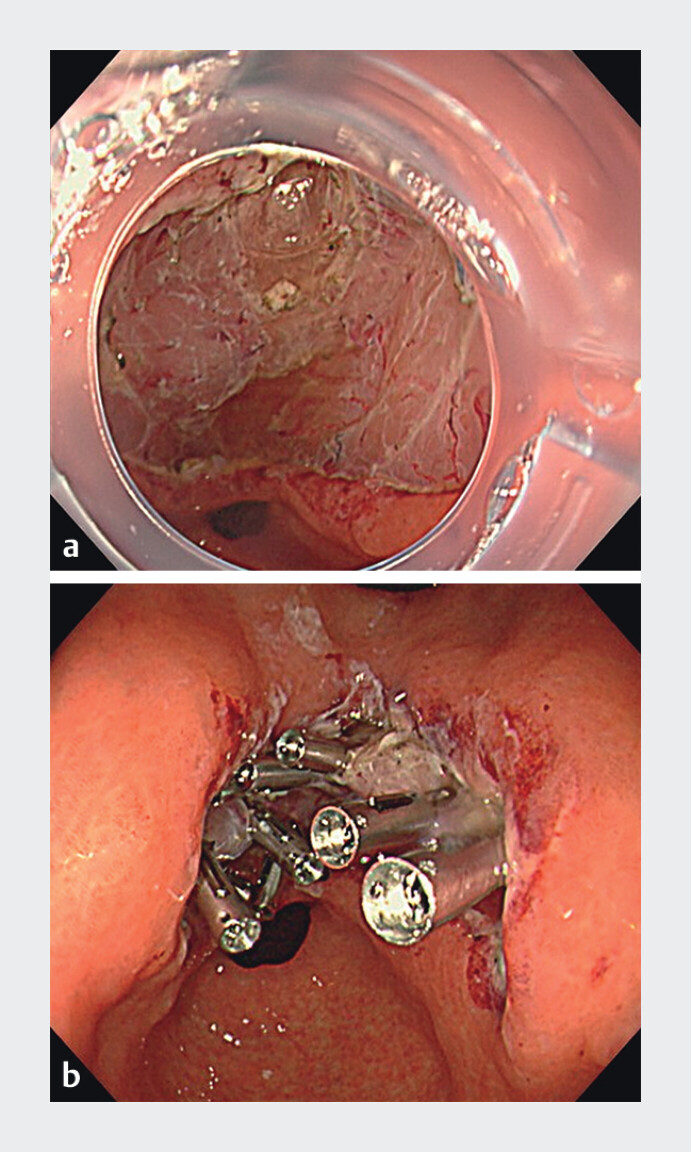
Condition after endoscopic submucosal dissection (ESD). **a** The ulceration followed gastric ESD in the lesser curvature of the gastric antrum. **b** After applying the search, coagulation, and clipping with the shrink method, the mucosal defect was effectively reduced and the ulcer base shrank to half its original size.

**Fig. 2:**
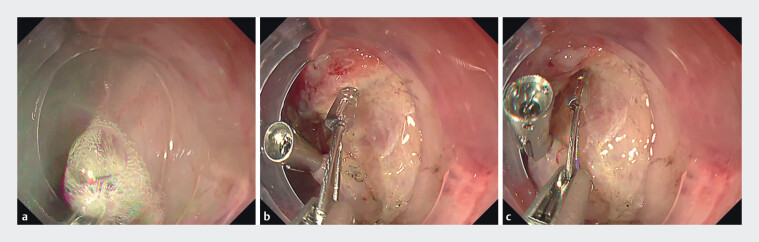
Search, coagulation, and clipping with the shrink method. **a** First, a coagulation procedure was performed after lesion resection, targeting vessels primarily at the margin of the ulcer base. **b,c** Subsequently, perforating vessels situated between the muscle layers were clipped using mainly 16-mm reopenable clips (SureClip; Micro-Tech Co. Ltd, Nanjing, China), while maintaining adequate suction to reduce luminal pressure.

Gastric ESD was performed on a 78-year-old woman classified as having an intermediate-risk of DB due to gastric antrum and tumor diameter of 30 mm. The ulceration followed gastric ESD in the lesser curvature of the gastric antrum ( Fig. 1 **a** ). Initially, coagulation was performed following lesion removal, with a focus on cauterizing vessels located mainly along the edge of the ulcer base ( Fig. 2 **a** ). Subsequently, perforating vessels situated between the muscle layers were clipped using mainly 16-mm reopenable clips, while maintaining adequate suction to reduce luminal pressure ( Fig. 2 **b** , Fig. 2 **c** ).

**Fig. 3:**
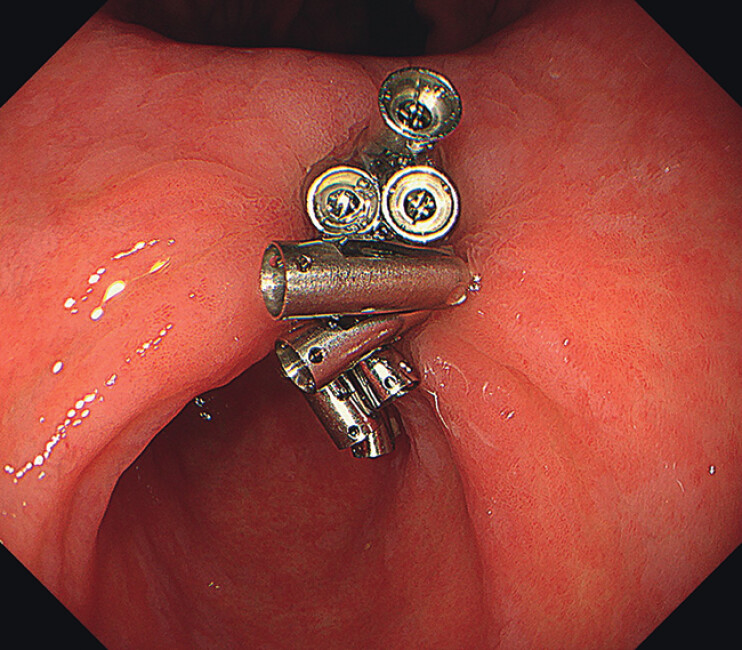
Follow-up endoscopy. After about 2 months, the ulcer was completely cured.

This Origami method 2 -inspired maneuver folded the muscle layer inward, effectively reducing the mucosal defect and shrinking the ulcer base to half its original size ( Fig. 1 **b** and Video 1 ). After about 2 months, the ulcer was completely cured ( Fig. 3 ).

The reopenable clips, which have been empirically found to have a stronger grasping force than the hemostatic clips (HX-610–135, Olympus Optical) previously used in the conventional SCC method 1 , are potentially less likely to dislodge. Therefore, they may be more effective in preventing DB. Several closure methods exist to prevent DB after gastric ESD 3 4 , but performing closure in all cases is time-consuming and costly. Given that low- and intermediate-risk patients account for nearly 90% in the BEST-J study 5 , the simpler and more cost-effective SCC-S method may be a reasonable option for these groups.
